# Rise and Shine: The Smoke-free Homes International Network (SHINE)

**DOI:** 10.18332/tpc/203919

**Published:** 2025-05-27

**Authors:** Rachel O’Donnell, Rebecca Howell, Piotr Teodorowski, Olena Tigova, Esteve Fernández, Sean Semple

**Affiliations:** 1 Institute for Social Marketing and Health, Faculty of Health and Sport Sciences, University of Stirling, Scotland, United Kingdom; 2 Tobacco Control Unit, Catalan Institute of Oncology – WHO Collaborating Centre for Tobacco Control, l’Hospitalet de Llobregat, Catalonia, Spain; 3 Centre for Biomedical Research in Respiratory Diseases, Institute of Health Carlos III, Madrid, Spain; 4 Tobacco Control Research Group, Bellvitge Biomedical Research Institute, l’Hospitalet de Llobregat, Catalonia, Spain; 5 Department of Clinical Sciences, Faculty of Medicine and Health Sciences, University of Barcelona, Barcelona, Spain; 6 Secretariat of Public Health, Department of Health, Generalitat de Catalunya, Barcelona, Spain

**Keywords:** smoke-free homes, secondhand smoke, SHINE, global health

As our international smoke-free homes network celebrates its 1st Birthday and reaches 100 members worldwide, we invite Tobacco Prevention and Cessation readers to join us (either by using this hyperlink or the QR code at the bottom of the Editorial) in working together to increase the rate at which non-smoking adults, and children, are protected from the harms posed by exposure to secondhand smoke (SHS) in the home.

SHINE’s overall goal is to encourage the World Health Organization (WHO) Framework Convention on Tobacco Control (FCTC) Conference of the Parties to modify Article 8 guidance to encourage signatory countries to include domestic settings as one of the key environments where non-smokers, particularly children, require protection from SHS exposure. The WHO FCTC requires that the 183 ratifying countries implement measures to protect people from exposure to tobacco smoke in public places, indoor workplaces, and public transportation. Whilst the WHO encourage the promotion of smoke-free homes^1^, there is currently no requirement to tackle SHS exposure in the home under the WHO FCTC, and yet this is where most exposure to SHS now occurs^2^.

To achieve this goal SHINE aims to:

- Connect researchers, practitioners and policy makers across the world, who are actively working to reduce SHS exposure in the home;
- Facilitate knowledge exchange and learning opportunities through webinars and newsletters; and
- Signpost members to smoke-free homes research, interventions and practical tools through our website.

## Why do we need SHINE?

By working together, tobacco control researchers, health practitioners, public health advocacy groups, and policymakers have achieved major global success in tackling the harms from breathing SHS. Over 2 billion people globally now live in countries where smoking is prohibited in most indoor public spaces. However, there is still a long way to go: SHS causes over 1 million premature deaths each year – twice the mortality that malaria produces each year^3^. SHS increases the risk of many diseases: living in a home with a smoker raises the risks of common diseases such as lung cancer, cardiovascular disease, stroke and respiratory disease by between 25–50%^4^. There is considerable variability in the prevalence of smoke-free homes between countries in Europe^5,6^. Evidence shows that there is also substantial inequality in exposure to SHS, with children living in poorer areas much more likely to suffer the harms of exposure to SHS at home in Europe^7^ and globally^8^. By effectively tackling smoking in homes, we can reduce one of the major causes of infant health inequalities globally.

Much of the existing research and regulatory focus around protecting non-smokers from SHS is on public spaces: from workplaces to transport to leisure settings. The Tobacco Control community has tended to consider the private space of the home as being too difficult to tackle in relation to SHS exposure. This is despite the fact that smoking in small, poorly ventilated spaces of the home generates some of the highest measured concentrations of SHS^9^. When these high concentrations are coupled to the amount of time people typically spend at home, it is quickly apparent that smoking in domestic settings is the key to reducing the health burden that SHS generates among non-smokers^10^. The SHINE network believes that increasing the proportion of smoke-free homes is an essential element of the tobacco endgame in the coming decade.

Children must be a particular focus in our effort to promote smoke-free homes. In addition to the direct health effects of SHS, children are also much more likely to become adults who smoke and experience the subsequent ill-health related to that smoking behaviour^11^. Protecting children from smoking in the home aligns squarely with the United Nations Convention on the Rights of the Child^12^. On this basis, some of our members have recently published a commentary which makes the case for a new approach to protecting children from tobacco smoke in the home^13^.

## What will SHINE seek to achieve?

SHINE will focus on four main areas of work that are key to reducing SHS exposure in the home globally.

*Mapping*

The network will work with our members to map existing international policies, examine existing metrics of progress, and identify what interventions are being used around the globe. Tobacco Control will grow stronger from learning what works in the fight to protect non-smokers from being exposed to SHS in their own home.

*Understanding*

We will seek to understand gendered differences in home smoking behaviors, through identifying who smokes in the home, and where and when smoking typically takes place. We can work together to understand the barriers to changing smoking behavior in homes and increase the levels of risk awareness.

*Framing*

As a community we will explore current understanding around options to create smoke-free homes including how to frame these options most effectively for policymakers and society.

*Communicating*

Via our network and through activities we will encourage the WHO to modify FCTC Article 8 guidance to include homes as one of the key environments where non-smoking adults and children require protection from SHS exposure.

Membership is open to researchers, practitioners and policy makers. We hope many of you will join us in achieving these goals and reducing the proportion of non-smokers who breathe SHS in their own home. It is time to rise and SHINE.

**Figure:**
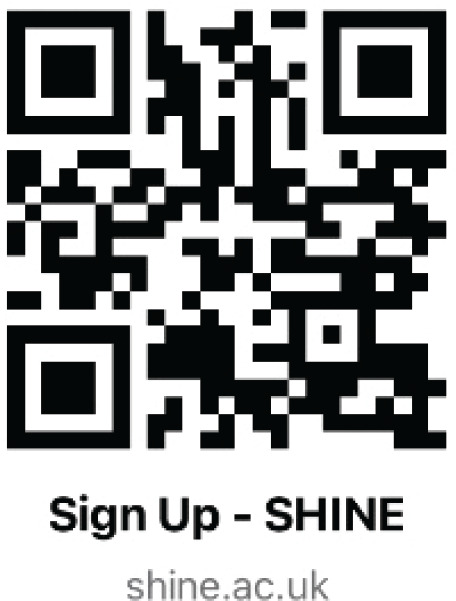


## Data Availability

Data sharing is not applicable to this article as no new data were created.
